# Human and mouse cortical astrocytes differ in aquaporin‐4 polarization toward microvessels

**DOI:** 10.1002/glia.23138

**Published:** 2017-03-20

**Authors:** Vigdis Andersen Eidsvaag, Rune Enger, Hans‐Arne Hansson, Per Kristian Eide, Erlend A. Nagelhus

**Affiliations:** ^1^ Department of NeurosurgeryOslo University Hospital, RikshospitaletOslo0027, Norway; ^2^ Institute of Clinical Medicine, Faculty of MedicineUniversity of OsloOsloNorway; ^3^ GliaLab and Letten Centre, Division of Physiology, Department of Molecular MedicineInstitute of Basic Medical Sciences, University of OsloOslo0317Norway; ^4^ Department of NeurologyOslo University Hospital, RikshospitaletOslo0027, Norway; ^5^ Department of Medical Biochemistry and Cell BiologyInstitute of Biomedicine, University of GothenburgGöteborgSweden

**Keywords:** AQP4, brain, electron microscopy, endfeet, perivascular

## Abstract

Aquaporin‐4 (AQP4), the predominant water channel in the brain, is expressed in astrocytes and ependymal cells. In rodents AQP4 is highly polarized to perivascular astrocytic endfeet and loss of AQP4 polarization is associated with disease. The present study was undertaken to compare the expression pattern of AQP4 in human and mouse cortical astrocytes. Cortical tissue specimens were sampled from 11 individuals undergoing neurosurgery wherein brain tissue was removed as part of the procedure, and compared with cortical tissue from 5 adult wild‐type mice processed similarly. The tissue samples were immersion‐fixed and prepared for AQP4 immunogold electron microscopy, allowing quantitative assessment of AQP4's subcellular distribution. In mouse we found that AQP4 water channels were prominently clustered around vessels, being 5 to 10‐fold more abundant in astrocytic endfoot membranes facing the capillary endothelium than in parenchymal astrocytic membranes. In contrast, AQP4 was markedly less polarized in human astrocytes, being only two to three‐fold enriched in astrocytic endfoot membranes adjacent to capillaries. The lower degree of AQP4 polarization in human subjects (1/3 of that in mice) was mainly due to higher AQP4 expression in parenchymal astrocytic membranes. We conclude that there are hitherto unrecognized species differences in AQP4 polarization toward microvessels in the cerebral cortex.

**Abbreviations**
AQP4: aquaporin‐4
EM: electron microscopic
CSF: cerebrospinal fluid
ISF: interstitial fluid
VRS: Virchow‐Robin paravascular space.

## 1 Introduction

The polarized distribution of AQP4 in the rodent central nervous system was discovered two decades ago (Nagelhus et al., 1998; Nielsen et al., 1997). Taking advantage of high resolution immunogold cytochemistry it was revealed that AQP4 water channels are clustered in astrocytic endfeet around blood vessels and along the pial surface (ibid.). Later it was shown that AQP4 polarization relies on AQP4 anchoring by the dystrophin‐associated protein complex (DAPC). Specifically, gene knockout studies in mice demonstrated that removal of dystrophin or other members of the DAPC reduces or abolishes AQP4 polarization to astrocytic endfoot membranes adjacent to pial or perivascular basal lamina (Amiry‐Moghaddam et al., 2003; Amiry‐Moghaddam et al., 2004; Enger et al., 2012; Frigeri et al., 2001; Lien et al., 2012; Neely et al., 2001; Noell et al., 2011). It was also shown that basal membrane laminin and agrin, which are ligands for the DAPC component dystroglycan, are necessary for AQP4 clustering in endfeet (Menezes et al., 2014; Noell et al., 2007; Rauch et al., 2011; Yao, Chen, Norris, & Strickland, 2014).

Accumulating evidence indicates that polarization of AQP4 is lost in rodent models for stroke (Frydenlund et al., 2006; Wang et al., 2012), traumatic brain injury (Liu et al., 2015; Ren et al., 2013), temporal lobe epilepsy (Alvestad et al., 2013), and Alzheimer's disease (Yang et al., 2011). The potential involvement of AQP4 in common neurological disorders and the discovery of AQP4‐dependent fluid and waste clearance (“glymphatic”) pathways (Haj‐Yasein et al., 2011; Iliff et al., 2012) have created regained interest in AQP4's distribution and function (Hladky & Barrand, 2014; Nagelhus & Ottersen, 2013; Papadopoulos & Verkman, 2013; Smith, Jin, & Verkman, 2015; Spector, Robert, & Johanson, 2015; Thrane, Rangroo, & Nedergaard, 2014; Thrane, Rangroo, Plog, & Nedergaard, 2015). Surprisingly, we still lack information on AQP4´s precise distribution in the human brain. One study included AQP4 immunogold data on hippocampal tissue resected from patients with mesial temporal lobe epilepsy (MTLE) and controls (Eid et al., 2005). However, the authors reported only relative changes in AQP4 expression and did not analyze the degree of AQP4 polarization. Because human cortical astrocytes are larger and structurally more complex than those of rodents (Oberheim et al., 2009), it is necessary to examine whether the subcellular distribution of AQP4 also differs in the two species. This study was undertaken to resolve this issue and includes brain tissue obtained from individuals without cerebrospinal fluid (CSF) related disorders. The human tissue specimens were obtained from neurosurgical operations where removal of brain tissue was required as part of the planned surgery, and processed similarly as brain tissue from adult wild‐type mice. Our study focused on astrocytic processes in the vicinity of vessels due to their involvement in glymphatic clearance of interstitial water and waste. We report that AQP4 signaling immunogold particles is less polarized towards microvessels in human than in mouse astrocytes since human parenchymal astrocytic membranes contain higher densities of AQP4 water channels.

## 2 Materials and methods

### 2.1 Study design and ethical approvals

The regional committee for medical and health research ethics (REK) of health region South‐East, Norway approved the human study: (i) brain tissue research Biobank (Approval no. REK 2010/1030 & 2012/1157). (ii) Storage of brain tissue from patients undergoing neurosurgery wherein brain tissue is removed as part of treatment (Approval no. REK 2011/2306). The human study was also approved by the Oslo University Hospital (Approvals no. 10/6806 and 2011/19311). Inclusion was by written and oral informed consent. The study on mice was approved by the animal care and use committee at the Institute of Basic Medical Sciences, University (FOTS #4309).

### 2.2 Study cohort

#### 2.2.1 Human brain tissue samples

Brain tissue samples were obtained from the following three groups of patients undergoing neurosurgery: (a) Individuals undergoing neurosurgery for epilepsy with brain tissue resection wherein a minor tissue sample was taken from the resected temporal lobe sample. The brain tissue specimens were collected from the superficial temporal cortical area, not from the sclerotic hippocampus. (b) Individuals undergoing neurosurgery with clipping of cerebral aneurysms in whom resection of tissue adjacent to the neck of the aneurysm was required. (c) Individuals undergoing neurosurgery for brain tumor in whom resection of brain tissue was required and a minor tissue sample was taken distant from the tumor in the resected sample.

The brain tissue biopsies were cut in three blocks of similar size and one of these blocks were immediately immersion fixated in 0.1 M phosphate buffer (pH 7.4) containing 4% paraformaldehyde and 0.25% glutaraldehyde. After 24 hr of fixation the blocks were stored in the same fixative diluted 1/10 in phosphate buffered saline until further processed.

#### 2.2.2 Mouse brain tissue samples

Five months old C57BL/6J (Charles River Laboratories, Sulzfeld, Germany) mice were deeply anesthetized with 4% isofluoran in a chamber. The mice were then decapitated and their brains were quickly removed. The brains were immediately cut in blocks of similar size as the human specimens, and handled the same way as described above for the human specimens.

### 2.3 Electron microscopic (EM) immunocytochemistry

Small blocks of the cortex were subjected to freeze substitution and infiltration in Lowicryl HM20 resin (Polysciences, Warrington, PA, Cat 15924) (Schwarz & Humbel, 1989). Using a Reichert ultramicrotome (Wien, Austria) sections of 80 nm thickness were cut, mounted on nickel grids and further processed for immunogold cytochemistry. Specifically, the sections were incubated in the following solutions: (1) 50 mM glycine in Tris buffer (5 mM) containing 0.01% Triton X‐100 and 50 mM NaCl (TBST; 10 min); (2) 2% human serum albumin (HSA) in TBST (10 min); (3) primary antibody (anti‐AQP4 from Sigma, 25 μg mL^−1^) diluted in the solution used in the preceding step (overnight); (4) same solution as in step 2 (10 min × 2); and (5) gold‐conjugated IgG (GAR15 nm for human sections and GAR10 nm for mouse sections, both obtained from Abcam), diluted 1:20 in TBST containing 2% HSA and polyethylene glycol (0.5 mg mL^−1^, 1 h). Sections were then counterstained.

### 2.4 Quantification of AQP4 immunogold distribution

The images were recorded with a Fei Tecnai 12 transmission electron microscope (FEI Company, Hillsboro, OR) at a nominal magnification of 43,000 in 2048 × 2048 × 8 bit. The images were acquired with analySIS image analysis software (Soft Imaging Systems GmbH, Münster, Germany). We used a version of the program that has been modified for semiautomatic evaluation of immunogold‐labeled membranes. Membrane segments of interest were drawn in the overlay and assigned a type label. Astrocytic endfeet were easily recognized due to their position next to vessels. The abluminal (i.e., facing neuropil) membrane of endfeet was chosen as the “parenchymal astrocytic membrane.” Gold particles in proximity to each membrane‐curve were detected semi‐automatically, and the distance between each particle's center of gravity and its membrane curve was calculated. Particles localized within ∼20 nm from their membrane curve were included in the automated calculation of the number of particles per unit length of membrane (linear particle density).

### 2.5 Statistics

The statistical analyses were performed using the SPSS software version 22 (IBM Corporation, Armonk, NY). Differences between continuous data were determined using independent samples *t* tests, while differences between categorical data were determined by Pearson Chi‐Square test. Correlation between variables was determined by the Pearson correlation coefficient. Statistical significance was accepted at the 0.05 level.

## 3 Results

### 3.1 Study cohort

The study cohort included 11 human subjects (age 22–73 years, mean 44 years) and 5 wild‐type mice (all 5 months) (Tables 1 and 2). In the 11 humans, the study material included astrocytic membrane domains in the vicinity of 33 brain capillaries (134 and 51 observations of astrocytic endfoot membranes facing endothelial cells and pericytes, respectively, and 124 observations of astrocytic endfoot membranes facing neuropil, i.e. “parenchymal astrocytic membranes”; Table 2) and 8 arterioles (80 observations of endfoot membranes facing the arteriole, 48 observations of parenchymal astrocytic membranes; Table 3). The mouse study included 26 cortical capillaries (95 and 47 observations of endfoot membranes facing endothelial cells and pericytes, respectively, and 92 observations of parenchymal astrocytic membranes; Table 2).

**Table 1:** Description of the human subjects included in the study

ID	Age (years)	Gender (F/M)	Diagnosis
1	36 y	F	Epilepsy
2	24 y	M	Epilepsy
3	54 y	F	Aneurysm
4	73 y	F	Tumor
5	40 y	F	Epilepsy
6	22 y	M	Epilepsy
7	25 y	F	Epilepsy
8	67 y	M	Aneurysm
9	46 y	M	Epilepsy
10	40 y	F	Epilepsy
11	58 y	F	Epilepsy

ID is the identity code of the human subjects.

**Table 2:** AQP4 immunogold labeling density along pericapillary and parenchymal astrocytic membrane domains in humans and mice

	**Capillaries (n)**	**Endfoot membrane facing endothelial cell**	**Endfoot membrane facing pericyte**	**Parenchymal membrane**	**Polarization towards endothelial cells (perivascular/parenchymal)**	**Polarization toward pericytes (perivascular/parenchymal)**
***Humans (ID)***						
3	2	31.2 ± 6.4 (4)	‐	16.1 ± 7.0 (4)	1.9	‐
4	9	20.9 ± 6.3 (34)	24.7 ± 6.4 (15)	6.3 ± 2.6 (42)	3.3	3.9
5	2	23.4 ± 3.6 (9)	23.1 ± 4.9 (5)	10.2 ± 4.0 (15)	2.3	2.3
6	3	26.8 ± 5.8 (12)	31.2 ± 2.5 (4)	17.2 ± 5.2 (12)	1.6	1.8
7	1	34.3 ± 5.3 (5)	35.9 ± 6.1 (7)	17.0 ± 5.3 (9)	2.0	2.1
8	2	23.7 ± 5.0 (9)	19.5 (1)	8.7 ± 3.0 (6)	2.7	2.2
9	4	27.7 ± 4.4 (17)	31.8 ± 5.3 (10)	14.2 ± 6.6 (14)	2.0	2.2
10	5	20.2 ± 5.5 (26)	23.5 ± 5.0 (5)	9.2 ± 3.4 (7)	2.2	2.6
11	5	20.8 ± 6.1 (18)	22.8 ± 4.6 (4)	11.0 ± 4.6 (15)	1.9	2.1
**Mean** ± **STD**		**25.4** ± **4.9**	**26.6** ± **5.7**	**12.2** ± **4.0**	**2.2** ± **0.5**	**2.4** ± **0.7**
***Mice***						
1	4	34.5 ± 7.9 (7)	32.1 ± 6.9 (13)	5.4 ± 6.7 (16)	6.4	6.0
2	4	27.5 ± 10.6 (17)	31.5 ± 10.0 (5)	3.0 ± 4.0 (21)	9.3	10.6
3	8	29.6 ± 10.5 (41)	31.7 ± 12.3 (12)	6.4 ± 5.6 (30)	4.6	5.0
4	2	36.4 ± 10.9 (10)	38.8 ± 10.4 (8)	6.7 ± 1.4 (5)	5.4	5.8
5	8	32.6 ± 7.8 (20)	32.9 ± 5.0 (9)	2.9 ± 3.6 (20)	11.4	11.5
**Mean** ± **STD**		**32.1** ± **3.6**	**33.4** ± **3.1**	**4.9** ± **1.8**	**7.4** ± **2.9**	**7.8** ± **3.0**

ID is the identity code of the human subjects. Data presented as mean ± STD with number of observations in parenthesis.

**Table 3:** AQP4 immunogold labeling density along astrocytic membrane domains in the vicinity of arterioles in human subjects

	**Arterioles (n)**	**Endfoot membrane facing arteriole**	**Parenchymal membrane**	**Polarization toward arteriole (endfoot/parenchymal)**
***Humans (ID)***				
1	3	16.5 ± 3.4 (17)	6.4 ± 3.1 (13)	2.6
2	3	22.4 ± 6.3 (34)	13.9 ± 6.2 (19)	1.6
7	1	24.6 ± 4.0 (16)	13.8 ± 7.9 (10)	1.8
11	1	20.7 ± 4.8 (13)	8.6 ± 2.7 (6)	2.4
**Mean** ± **STD**		**21.1** ± **3.4**	**10.7** ± **3.8**	**2.1** ± **0.5**

ID is the identity code of the human subjects. Data presented as mean ± STD with number of observations in parenthesis.

### 3.2 Subcellular distribution of AQP4 immunogold labeling in humans and mice

**Figure 1:**
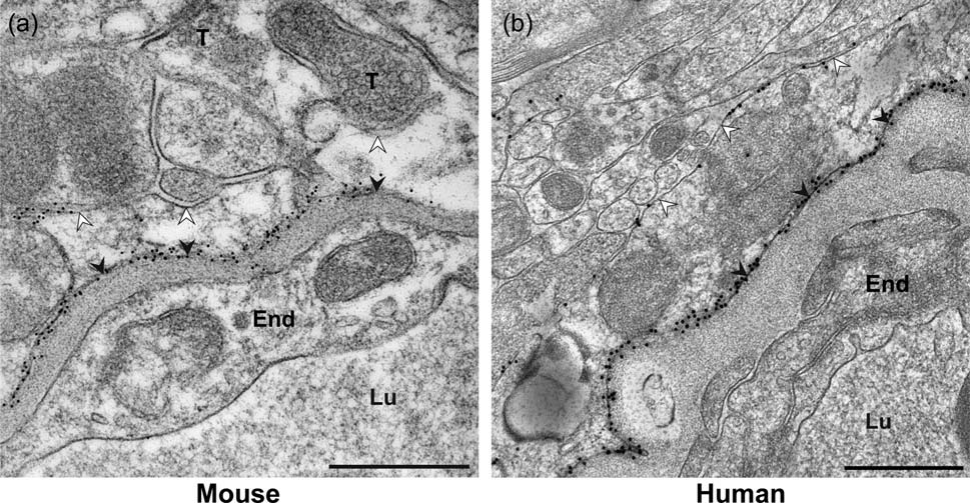
AQP4 distribution in the mouse and human cerebral cortex revealed by immunogold cytochemistry. The human tissue was removed from the ID 7 subject (see Table 1) as part of neurosurgical treatment of epilepsy. Representative micrographs from (a) mouse and (b) human showing AQP4 labeling over astrocytic membranes in the vicinity of a capillary. The astrocytic endfoot membranes facing the neuropil (abluminal or “parenchymal astrocytic membrane”, cf. Materials and Methods) and endothelium (adluminal membrane) are indicated by white and black arrowheads, respectively. The AQP4 immunogold particles are concentrated in the adluminal endfoot membranes in both species, but AQP4 polarization is clearly less in human than in mouse. The mouse micrograph shows that segments of the parenchymal astrocytic membrane ensheathe nerve terminals (T). End, capillary endothelium; Lu, capillary lumen; RBC, red blood cell. Scale bars, 0.5 μm

**Figure 2:**
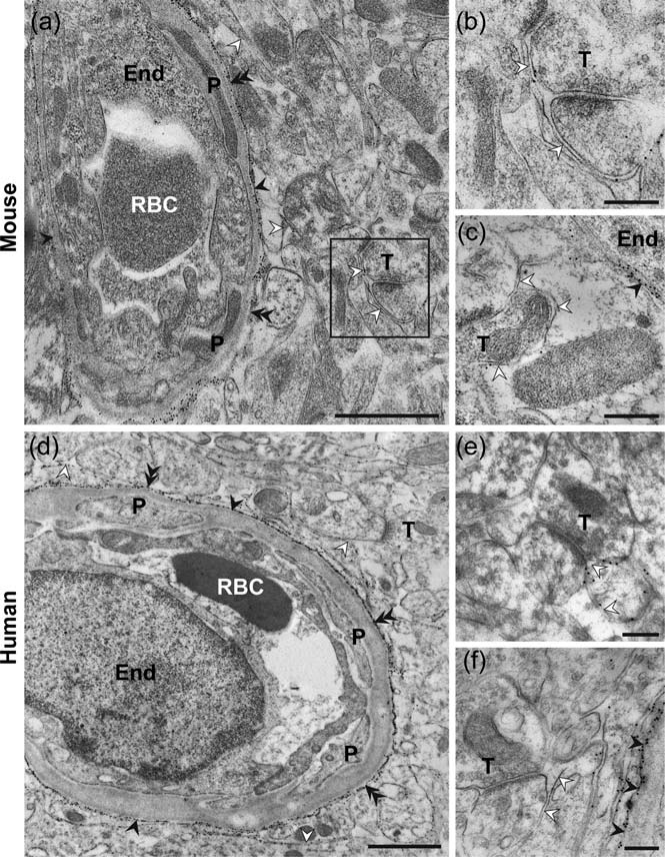
AQP4 immunogold labeling in the mouse (a–c) and human (d–f) cerebral cortex. (d) and (f) are from subject ID 4, whereas (e) is from ID 5 (see Table 1). Both adluminal endfoot membranes and perisynaptic astrocytic membranes are indicated with open arrowheads. Double filled arrowheads denote astrocytic endfoot membrane segments adjacent to pericytes (P). Other labels are as in Figure 1. Boxed region in A is shown at higher magnification in (b). Scale bars, (a, d): 1 μm; (b, c, e, f): 0.25 μm

**Figure 3:**
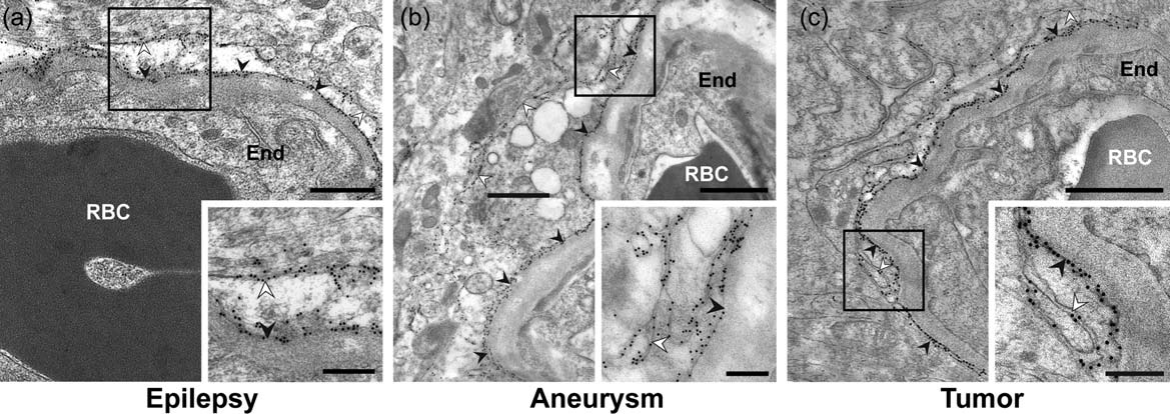
AQP4 immunogold labeling of human cortical specimens. The tissue sections were obtained from subjects referred for neurosurgical treatment of (a) epilepsy (ID 6, see Table 1), (b) aneurysm (ID 3), and (c) tumor (ID 4). Labels as in Figure 1. Boxed areas are shown in insets. Scale bars, 1 μm and 0.5 μm (inset)

**Figure 4:**
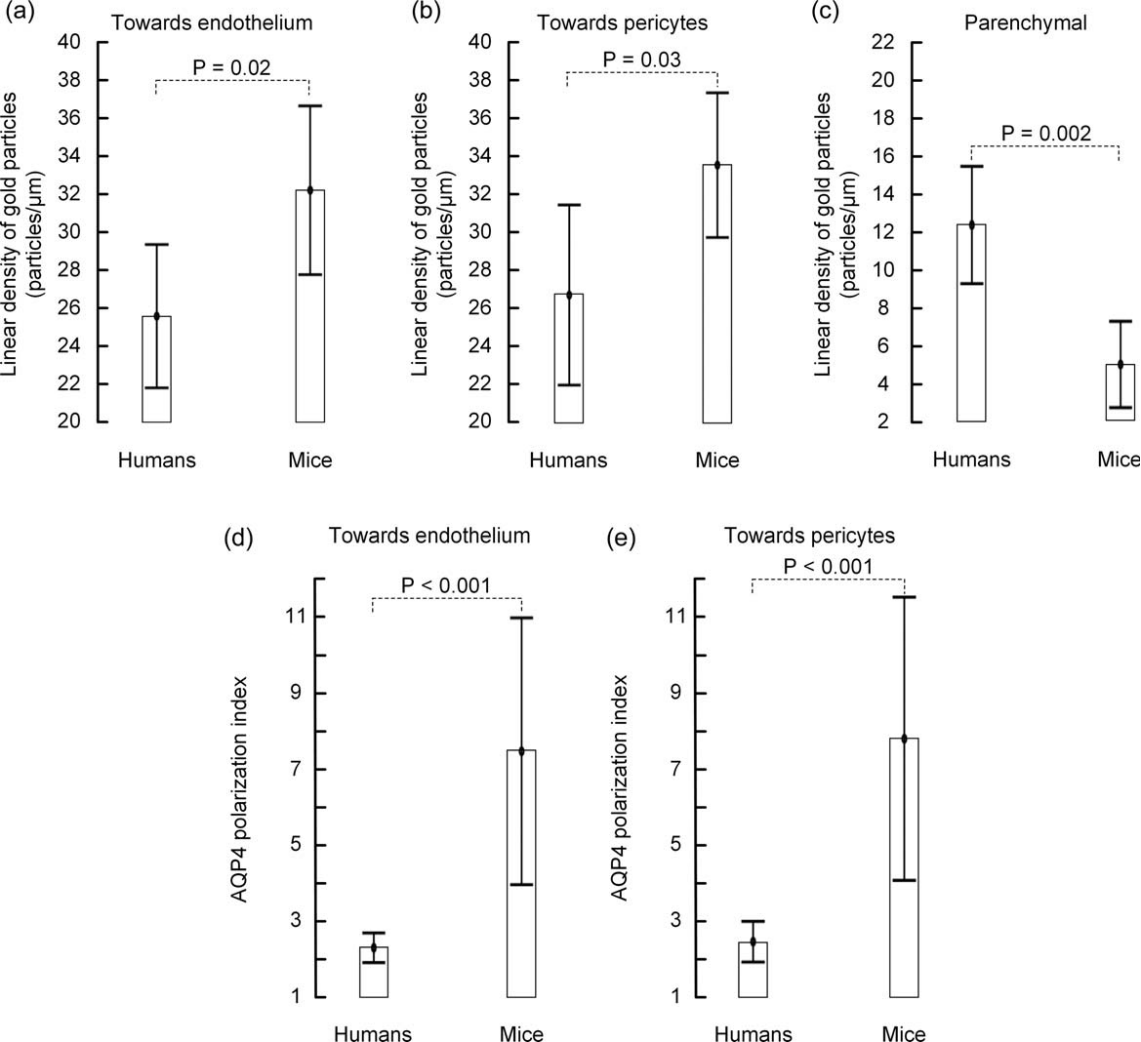
Quantitative analysis of AQP4 immunogold labeling in humans and mice. Comparison of linear densities of AQP4 signaling gold particles along (a) the astrocytic endfoot membrane towards the capillary endothelium, (b) the astrocytic endfoot membrane towards pericytes, and (c) the parenchymal astrocytic membrane (facing neuropil) in humans and mice. (d) AQP4 polarization towards endothelial cells, that is, the ratio of AQP4 immunogold labeling density over membranes facing the endothelium and neuropil, in humans versus mice. (e) AQP4 polarization towards pericytes. Values are mean with 95% confidence interval, and differences between the species were determined by independent samples *t* test. The degree of polarization towards both endothelial cells and pericytes was significantly higher in mouse (d–e)

AQP4 signaling gold particles were concentrated along astrocytic endfoot membranes facing capillary endothelial cells in both mouse (Figure 1a) and human (Figure 1b) specimens. Endfoot membrane domains adjacent to pericytes displayed similar AQP4 labeling as those facing the endothelium (Figure 2a,d). Furthermore, the AQP4 labeling over the abluminal membranes of pericapillary endfeet was comparable to that over astrocytic membranes elsewhere in the neuropil, including those next to synapses (Figure 2a–f). The AQP4 immunogold labeling pattern was similar in human tissue resected from subjects with epilepsy, aneurysm and tumor (Figure 3a–c). Quantitative analysis revealed that the linear density of gold particles along endfoot membranes facing the capillary endothelium was somewhat higher in mice than in human subjects (Figure 4a; Table 2), likely reflecting the more sensitive immunostaining protocol used in mice (see Discussion). Compared with humans mice also displayed higher density of gold particles along endfoot membranes adjacent to pericytes (Figure 4b; Table 2).

### 3.3 Polarization of AQP4 to perivascular astrocytic endfeet

When comparing the ratio of AQP4 labeling along astrocytic endfoot membranes facing capillary endothelial cells (Figure 4a) or pericytes (Figure 4b) versus that along parenchymal astrocytic membranes (Figure 4c), i.e., the AQP4 polarization index, we found prominent differences between mice and humans (Figure 4d,e; Table 2). Specifically, whereas AQP4 immunogold labeling density along membranes facing the endothelium was 7.4 ± 2.9 (range 4.6–11.4) fold higher than along parenchymal astrocytic membranes in mice, the corresponding factor in humans was only 2.2 ± 0.5 (range 1.6–3.3). Similar species differences were found for AQP4 polarization towards membranes adjacent to pericytes. The fact that the AQP4 polarization index in humans was only 1/3 of that in mice mainly relied on species differences in AQP4 expression in parenchymal astrocytic membranes (Figure 4c; Table 2). Notably, the AQP4 immunogold density along parenchymal membranes was ∼2.5 fold higher in humans than in mice, despite that a less sensitive immunostaining protocol was used for the human tissue sections (see Discussion).

**Figure 5:**
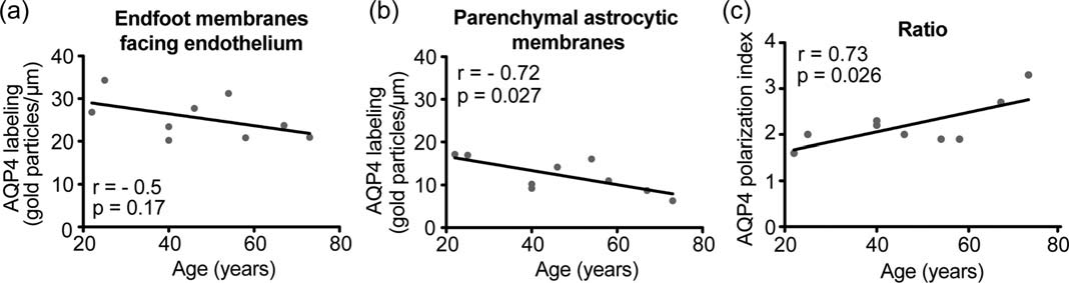
Correlation between age of the human individual and AQP4 immunogold labeling. Plots of age versus AQP4 labeling along (a) endfoot membranes next to capillary endothelial cells, or (b) parenchymal astrocytic membranes in the vicinity of capillaries. (c) Plot of age versus AQP4 polarization towards capillaries, that is, the ratio of AQP4 immunogold labeling density along the two membrane domains in (a) and (b). The Pearson correlation coefficient (r) and significance level is given for each plot

Because our study included human individuals of different age we analyzed whether AQP4 immunogold labeling varied with age. AQP4 labeling in endfoot membranes next to capillary endothelial cells was independent of age (Figure 5a). A statistically significant negative correlation with age was found for the AQP4 expression in parenchymal astrocytic membranes (Figure 5b), causing AQP4 polarization to increase somewhat with age (Figure 5c).

**Figure 6:**
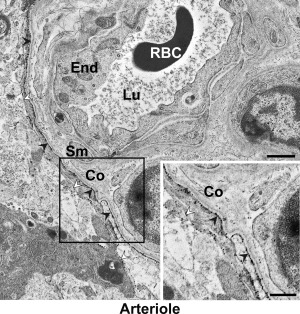
AQP4 immunogold labeling in the vicinity of a human arteriole. The specimen is from subject ID 1. The density of AQP4 signaling gold particles is higher in the endfoot membrane facing the arteriole (filled arrowheads) than in the membrane facing neuropil (open arrowheads). Note collagen (Co) fibers in the para‐arteriolar space. Sm, vascular smooth muscle cell. Other legends as in Figure 1. Scale bars, 1 μm and 0.5 μm (inset)

**Figure 7:**
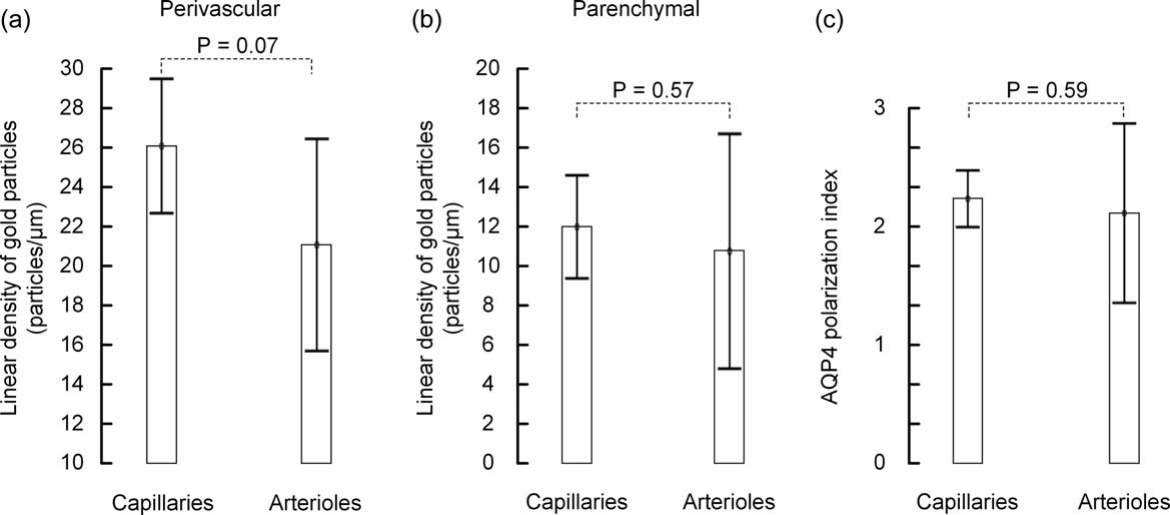
AQP4 immunogold labeling around human capillaries versus arterioles. Comparison of the two vessel segments with regard to (a) linear density of gold particles over the perivascular astrocytic endfoot membrane (endfoot membranes facing capillary endothelial cells and pericytes were pooled), (b) linear density of gold particles over the parenchymal astrocytic endfoot membrane (facing neuropil), and (c) AQP4 polarization index (ratio of labeling over perivascular versus parenchymal astrocytic membranes). Values are mean with 95% confidence interval, and differences between the species were determined by independent samples *t* test. There were no significant differences between peri‐capillary and peri‐arteriolar AQP4 labeling patterns.

In human subjects we also compared AQP4's distribution in astrocytic membranes around arterioles versus capillaries. As pericapillary endfoot membranes we included both membranes facing endothelial cells and pericytes. Neither endfoot nor parenchymal AQP4 immunogold labeling densities differed for the two vessel segments (Figures 6 and 7; Tables 2 and 3). Hence, the degree of AQP4 polarization was similar for arterioles and capillaries.

## 4 Discussion

The major findings in this study were (1) a higher AQP4 immunogold labeling density over parenchymal astrocytic membranes in human subjects than in mice, and (2) a lower degree of AQP4 immunogold polarization to perivascular astrocytic endfoot membranes in humans compared with mice. In the human individuals, endfoot AQP4 polarization did not differ between capillary and arteriolar vessel segments.

Immunogold cytochemistry is a powerful method to quantify the density of molecules along specific membrane domains and in cellular subcompartments (Amiry‐Moghaddam & Ottersen, 2013). The immunogold demonstration of AQP4 clustering in glial endfoot membranes (Nagelhus et al., 1998; Nielsen et al., 1997) unveiled that rodent glia—contrary to common belief at the time—are highly polarized, molecularly and functionally. Except one study (Eid et al., 2005), we are not aware of studies exploring AQP4 expression in the human brain by this technique.

We aimed at studying human individuals without CSF circulation disturbances with brain tissue specimens from apparently healthy brain. Because it is not possible to obtain brain tissue from healthy individuals, we gathered brain tissue from individuals undergoing elective neurosurgery for various reasons (epilepsy, brain tumor, or cerebral aneurysm), in whom resection of normal brain tissue was required for the planned surgery. Eight out of eleven patients had epilepsy. This could potentially influence our results as certain types of epilepsy affect AQP4 polarization. Notably, loss of AQP4 polarization was found in sclerotic hippocampi from humans with MTLE (Eid et al., 2005) and in the kainate model of the same condition (Alvestad et al., 2013). However, in MTLE AQP4 polarization was reduced by loss of AQP4 from endfoot membranes (Eid et al., 2005). Alvestad et al. (2013) found that the AQP4 expression in parenchymal astrocytic membranes was stable or only slightly increased. In contrast, the low AQP4 polarization index of human subjects in our study relied on relatively high AQP4 expression in parenchymal astrocytic membranes. Furthermore, the tissue investigated in the present study was sampled from the temporal lobe and not from sclerotic hippocampi. The fact that AQP4 polarization was similar in all human subjects and independent of the cause of neurosurgical intervention (epilepsy, tumor, aneurism), argue for that the tissue specimens analyzed were representative for the healthy human brain. We can, however, not rule out the possibility that disease has influenced AQP4's distribution our human samples.

Could the differences in AQP4 polarization between the two species rely on differences in maturity or age‐related changes? It is well established that AQP4 expression in brain increases from birth until adulthood (Lunde et al., 2015; Wen et al., 1999). However, the AQP4 expression around cerebral capillaries in C57BL/6J mice is stable from young adulthood (2–3 months) to senescence (18–20 months) (Kress et al., 2014). Only brain tissue immediately surrounding penetrating cortical arterioles show increased AQP4 expression in old mice, possibly due to astrogliosis (ibid.). In our study we used 5 month C57BL/6J mice whereas the human subjects ranged from 22 to 73 years (mean 44 years). C57BL/6J mice of 3–6 months are mature adults corresponding to human age 20–30 years (Flurkey & Currer, 2004). Even though only 3 of the 11 human individuals matched the age of the mice, the findings of Kress et al. indicate that inclusion of older mice would not have changed our conclusions. It should also be noted that none of the human individuals presented an AQP4 polarization index within the range found in mice.

Neither in our human cohort did AQP4 expression in pericapillary endfoot membranes vary with age of the individual. However, a statistically significant negative correlation with age was found for AQP4 expression in parenchymal astrocytic membranes, causing AQP4 polarization to *increase* somewhat with age for humans.

Are AQP4 immunogold data from immersion‐fixed tissue representative for AQP4's distribution in the intact brain? It has been reported that the subcellular distribution of AQP4 is sensitive to ischemia (Frydenlund et al., 2006). However, significant changes in cortical AQP4 expression did not occur until 24 hr after temporary (90 min) middle cerebral artery occlusion and involved loss of AQP4 from perivascular endfoot membranes (ibid.). Our tissue specimens were immersion‐fixed within minutes. The fact that our mouse AQP4 immunogold data are similar to previously published data from perfusion‐fixed mouse brain (Amiry‐Moghaddam et al., 2003; Enger et al., 2012) indicates that AQP4's distribution is not artifactually altered during the immersion‐fixation procedure.

The human and mouse specimens were subjected to identical fixation and freeze‐substitution embedding protocols. However, the immunogold labeling protocol used for mouse sections included EMGAR10 gold‐conjugated secondary antibodies which are more sensitive than the EMGAR15 antibodies used for human sections (Mathiisen et al., 2006). Thus, despite that the AQP4 immunogold density along perivascular endfoot membranes was higher in mice than in human subjects, we cannot conclude that there are species differences in AQP4 expression levels in these membrane domains. However, we can conclude that the parenchymal astrocytic membranes in humans have higher AQP4 expression than those in mice, since the former displayed higher AQP4 immunogold densities despite being labeled with the least sensitive gold‐conjugated secondary antibodies. It should be emphasized that the ratio of immunogold densities along different membrane domains within the same section is independent of the size of the gold nanoparticles, as long reliable immunostaining signals are achieved. As both EMGAR15 and EMGAR10 gave robust immunogold labeling, we conclude that there are consistent and prominent species differences in AQP4 distribution and polarization.

We did not quantify AQP4 labeling in other parenchymal membrane domains than those belonging to astrocytic endfeet. The main reason for this was that astrocytic endfeet were easily recognized due to their position next to vessels. Many processes elsewhere in the parenchyma were hard to classify due to the suboptimal morphology of immersion‐fixed tissue treated with immunoreagents.

Does the abluminal membrane segment of pericapillary endfeet have the same AQP4 expression as astrocytic membranes around synapses and elsewhere in the parenchyma? The only study addressing this question dealt with the retina and found comparable AQP4 immunogold labeling densities in all parenchymal glial membrane domains analyzed, including those ensheathing synapses and abluminal segments of endfeet (Nagelhus et al., 1998). The clustering of AQP4 in endfoot membranes adjacent to vessels is due to AQP4 anchoring by the DAPC, which binds laminin and agrin in the perivascular basal lamina. Because no other AQP4 anchoring mechanisms have been identified, it is likely that AQP4 water channels diffuse freely in parenchymal glial membranes and distribute uniformly. However, different types of astrocytes may have different levels of AQP4, as pointed out below.

Why are AQP4 water channels highly polarized to perivascular astrocytic endfeet in rodents (Nagelhus et al., 1998; Nielsen et al., 1997), while showing less polarization in man? It is tempting to relate the species differences in AQP4 distribution to differences in brain size and astrocyte morphology. The human brain is much larger and more complex than that of rodents and the number of astrocytes per neuron in the human cortex is exceeding that in rodents (Verkhratsky & Butt, 2013). Furthermore, human astrocytes have in the order of 3 times greater dimensions than rodent astrocytes and are further characterized by more extensively branching processes (Oberheim et al., 2009; Pekny & Pekna, 2016; Verkhratsky & Butt, 2013). A unique feature of the human brain is the presence of astrocytes with long processes that span several cortical layers and traverse the domain of other astrocytes (Oberheim et al., 2009; Sosunov et al., 2014). Long‐process astrocytes express the extracellular matrix receptor CD44, whereas protoplasmic astrocytes are CD44 negative. The cell bodies of CD44 positive long‐process astrocytes are typically found underneath the pia mater, but also locate around large blood vessels and in deep cortical layers. A peculiar finding is that long‐process astrocytes seemingly display higher AQP4 immunofluorescence staining around cell bodies and in parenchymal processes than do protoplasmic astrocytes (Sosunov et al., 2014). Furthermore, long‐process astrocytes also contact blood vessels, but with smaller endfeet than protoplasmic astrocytes (ibid.). Because we selected all endfeet that abutted the vessels, long‐process astrocytes could also be included in our analyses. However, the vast majority of processes must have belonged to protoplasmic astrocytes as they cover most of the vascular surface.

A previous study in mice advocated that pericytes guided AQP4 polarization (Gundersen, Vindedal, Skare, & Nagelhus, 2014). In humans, however, we found no difference in AQP4 immunogold density along endfoot membranes facing endothelial cells and those facing pericytes. This illustrates another species difference between rodents and humans.

What are the functional implications of the rather high AQP4 content in parenchymal astrocytic membranes in the human brain? Despite two decades of research using various rodent models there is no consensus on the driving forces for AQP4 mediated water transport in brain (Assentoft, Larsen, & MacAulay, 2015; Zhang & Verkman, 2008). Many physiological functions have been attributed to AQP4 (Nagelhus & Ottersen, 2013; Papadopoulos & Verkman, 2013), including extracellular space volume regulation (Haj‐Yasein et al., 2012; Yao, Hrabetova, Nicholson, & Manley, 2008) and drainage of brain interstitial water (Haj‐Yasein et al., 2011). A recent breakthrough came with the discovery that glial AQP4 also facilitates clearance of brain interstitial waste along paravascular glymphatic pathways (Iliff et al., 2012). However, the glymphatic concept, which involves convective fluid transport from para‐arterial to paravenous spaces through the brain extracellular space, is controversial. Notably, the mechanistic underpinning of brain interstitial solute movement and the direction of fluid flow along vessels are disputed (Asgari, de Zélicourt, & Kurtcuoglu, 2016; Smith et al., 2015; Spector et al., 2015; Tarasoff‐Conway et al., 2015). Modeling studies even argue for substantial intracellular water movement (Asgari, de Zélicourt, & Kurtcuoglu, 2015) and fail to support that convective flow moves solutes through brain extracellular space at physiologically relevant pressures (Jin, Smith, & Verkman, 2016). Whatever the driving forces and exit pathways, knowing the exact distribution of AQP4 water channels in astrocytic membranes is critical for our understanding of brain water movement and waste clearance in health and disease.

## 5 Conclusions

Our comparative study provides evidence that human cortical astrocytes contain several‐fold higher density of AQP4 water channels in their parenchymal membranes than do mouse cortical astrocytes, whilst endfoot membranes covering blood vessels display similar AQP4 levels. The fact that AQP4 polarization in humans is 1/3 of that in mice must be taken into consideration when translating physiological and pathophysiological findings in rodents to humans in a clinical setting.
